# The role of children and their socioeconomic resources for the risk of hospitalisation and mortality – a nationwide register-based study of the total Swedish population over the age 70

**DOI:** 10.1186/s12877-019-1134-y

**Published:** 2019-04-23

**Authors:** Anna C. Meyer, Hannah L. Brooke, Karin Modig

**Affiliations:** 1 Unit of Epidemiology, Institute of Environmental Medicine, Karolinska Institutet, PO Box 210, SE-171 77 Stockholm, Sweden; 2 Department of Public Health and Caring Science, Uppsala Universitet, 751 22 Uppsala, Sweden

**Keywords:** Parity, Social support, Old age

## Abstract

**Background:**

Previous studies have shown that mortality in old age is associated with both number of children and their socioeconomic resources. The underlying mechanisms are unclear, as well as when during the process of health deterioration the advantage of parents over non-parents arises. This study aims to examine how the number of children and their socioeconomic resources are associated with different health outcomes among their parents, namely the hazard for i) first hospitalisation, ii) re-admission, iii) mortality after first hospitalisation, and iv) overall mortality.

**Method:**

This longitudinal cohort study includes all individuals born 1920–1940 who were living in Sweden at age 70 years (890,544 individuals). Individuals were linked to their offspring and spouse using administrative registers and followed for up to 25 years. Associations were estimated using multivariable Cox models adjusted for index persons’ education and income, marital status, their partners’ education, and age at first birth.

**Results:**

In this study, having children was associated with reduced mortality risk of their parents, but not with the risk of being hospitalised, which increased as number of children increased. A higher education of children was protective for all parental outcomes independent of number of children and their financial resources. In fact, income of the children was only weakly associated with the health of their parents.

**Conclusions:**

The benefit of having children compared to childlessness for health in old age seems to arise once individuals have become ill rather than before. Children’s education is important for parental health and mortality, in fact more important than the number of children itself in this Swedish cohort.

**Electronic supplementary material:**

The online version of this article (10.1186/s12877-019-1134-y) contains supplementary material, which is available to authorized users.

## Background

It is well-established that childless individuals and parents with many children experience higher mortality than those with few children [1–5], but mechanisms behind this finding are less clear. Both biological [3, 6, 7] and social mechanisms have been proposed to explain the associations between parity and mortality [4]. Social mechanisms refer to social influence on health behaviours as well as different kinds of support that adult children can provide, potentially affecting the health and survival chances of their parents. The support that adult children can provide to their ageing parents can be both emotional, informational, and instrumental and has been hypothesised to be particularly important in old age when health starts to deteriorate [4]. Older childless individuals may thus face support deficits when in poor health and living alone and/or lacking family support [8, 9].

In studies of parity and survival, health related selection is often argued to explain part (or all) of the survival advantage of parents over non parents [3, 6, 7]. That is, parents are in general healthier than non-parents, and parents of two children are often healthier and more highly educated than parents of many children. However, the absolute difference in mortality between parents and childless individuals has shown to increase with age [10], and to be largest in ages above 90 years. If health selection was to explain all of the differences in health between parents and non-parents, we would not expect the differences to grow larger in ages well beyond average life expectancy when selection generally has played out its role. Previous research has also shown that adult children often become care-givers for their aging parents [11].

It is yet unclear when during the process of health deterioration the advantage of parents over non-parents arises, if support form adult children matters for their parents risk of developing diseases, for the chance of surviving them, or both. Previous studies examining the role of children for parental health have not systematically distinguished onset of ill health from survival after ill health has begun. Separating this is important in order to understand the advantage of parents over non-parents, and may to some extent help to disentangle the two proposed mechanisms, selection and support. For example, while health selection into parenthood or parity may play a role for differences in the occurrence of disease or overall mortality, it is less likely that selection explains disparities in survival once a disease has occurred. Instead, it can be hypothesised that structural support has a larger impact for the prognosis once a disease has manifested. Without arguing for one of these mechanisms (selection vs. support) before the other, this paper aims to describe differences in health at different stages between childless and parents.

In addition to the mere presence of children, studies have suggested that the socioeconomic resources of children are inversely related to their parent’s mortality, independent of parents’ own socioeconomic resources [12–18]. Even though the causal directions are hard to disentangle (since socioeconomic disadvantage or compromised health of parents might negatively affect educational attainment among offspring), there is some evidence for a causal benefit at least of daughters’ education on their fathers’ mortality [19]. Further, a previous study has shown that a higher education level among children is positively associated with mothers’ survival after a breast cancer diagnosis [20]. One explanation could be greater health knowledge among highly educated children, who are better placed to communicate about health behaviours, help their parents to navigate complex healthcare systems, with compliance to medication or rehabilitation programs, or seek the best available care for an ill parent [12, 14, 21]. Less is known about the association between children’s financial resources and parental health, but two previous studies found children’s education to be more closely related to mortality in old age than their income [22, 23].

In this study we aim to examine how the number of children as well as children’s socioeconomic resources are associated with their parents’ risk of hospitalisation (which may be seen as a proxy for onset of ill health), risk of re-admission (i.e. prognosis/recurrence of disease), and mortality after hospitalisation (i.e. survival after ill health has begun) as well as overall mortality in older adults.

## Methods

### Study population

This population-based cohort study includes all individuals born 1920–1940 alive and residing in Sweden at age 70. These individuals were followed from January 1st of the year of their 70th birthday until emigration, death, or the end of follow-up (December, 31st 2014). Information about hospitalisations was retrieved from the National Patient Register. Data on socioeconomic variables were obtained from the Longitudinal Integration Database for Health Insurance and Labour Market Studies or from the population and housing census in 1970. Family members were linked through the Multi-Generation Register.

### Outcome variables

The four outcomes of interest were first hospitalisation, re-admission, mortality after hospitalisation and overall mortality. Hospitalisation was defined as the first hospitalisation in the National Patient Register after age 70 with a length of at least 2 nights regardless of diagnosis. Individuals who had been hospitalised during 5 years before study entry were excluded from the study population. Re-admission was defined as a subsequent hospitalisation of at least 2 nights.

### Exposure variables

The number of adult children was calculated as the total number of biological or adopted children aged ≥25 years, alive and registered in Sweden. This age criterion was set to allow children to reach a stable socioeconomic position. Number of children was categorised as 0, 1, 2, 3, 4, and ≥ 5 with one child as reference group. Adult children’s education level was categorised as: 1) basic education (i.e. ≤9 years of compulsory education; reference group); 2) ≤2 years of secondary education; 3) > 2 years of secondary education; 4) ≤2 years of tertiary education; and 5) > 2 years of tertiary education. Adult children’s income level when their parents were aged 69 years was calculated as the pooled income of all household members, including labour and capital incomes and social transfers, divided by the household consumption weights provided by Statistics Sweden^1^ [24]. Income was categorised into quintiles for each year. This measure reflects the relative income level, which accounts for population level trends in absolute income over time [23]. We did not include income as a time-varying factor since this would require the assumption that a child’s income influences parental hospitalisation and mortality immediately. Instead we find it reasonable to assume that the relative income quintile will remain more or less stable over time. If parents had more than one child, the highest education or income level among children was used [23]. All exposure variables and covariates were measured at the end of the year before study entry.

### Covariates

Individuals were categorised as married or unmarried, as the presence of a partner may impact health. The unmarried category contained never married, widowed, and divorced individuals. Education of index persons and their partners was divided into basic education (≤9 years of compulsory education), secondary education (≤12 years of education); and tertiary education (> 12 years of education), since higher education is rare in these birth cohorts. Income of index persons was calculated in the same way as for adult children but income quintiles were calculated separately for parents and children to account for a retirement effect on income.

Since maternal age at first birth is correlated with parity and associated with mortality independent of number of children [25], parental age at first birth was considered as confounder. This was categorised as < 20 years, 20–24 years, 25–29 years, 30–34 years, 35–39 years, and ≥ 40 years.

### Statistical analyses

First, the associations between number of adult children and parental morbidity and mortality were analysed, including also those with 0 children. Next, children’s socioeconomic resources and parental morbidity and mortality were analysed among parents. Hazard Ratios (HR) and 95% confidence intervals (CI) for each of these outcomes were estimated using multivariable Cox proportional hazards regression with age as the underlying time scale. The proportional hazards assumption was examined using scaled Schönfeld residuals and log-log plots. Baseline hazards were allowed to vary by marital status and by either birth cohort or year of first hospitalisation. Survival proportions were estimated at age 80 and 87 based on adjusted models with covariates held constant at their means or the most common category. Similarly, we estimated the proportion of individuals not hospitalised by age 80, the proportion not re-admitted 2 years after first hospitalisation, and the proportion surviving 5 years after first hospitalisation. All analyses were stratified by sex and conducted using Stata version 14 (StataCorp LP, College Station, TX, USA).

## Results

The study population included 890,544 individuals, 45% male and 55% female (Table 1). In total, 735,229 hospitalisations (most often for ischemic heart disease, arthrosis and cerebrovascular diseases) and 384,154 deaths occurred during the follow-up. The proportion of childless individuals was 20% for men and 15% for women.

**Table 1:** Characteristics of study population by sex and number of children (*N* = 890,544)

Number of children	Men (*n* = 401,997)						Women (*n* = 488,547)					
	0 *n* = 79,653	1 n = 79,268	2 *n* = 142,256	3 *n* = 68,807	4 *n* = 21,905	≥5 *n* = 10,108	0 *n* = 75,236	1 *n* = 103,136	2 *n* = 174,801	3 *n* = 88,242	4 *n* = 30,919	≥5 *n* = 16,213
Median age at death (years)	83.90	85.36	86.04	85.84	85.12	84.13	87.46	88.16	88.82	88.67	88.09	87.23
Proportion who died (%)	50.7	44.7	37.6	39.3	45.2	53.2	39.5	36.4	29.7	30.7	35.2	42.6
Median age at first hosp. (years)	75.88	75.98	76.10	75.85	75.45	75.17	77.50	77.34	77.40	77.01	76.58	76.22
Proportion with hospitalisation (%)	72.5	73.9	71.0	72.8	76.5	79.1	70.6	72.4	68.8	70.3	74.4	78.7
Mean age at first birth (years)	–	30.24	27.88	26.23	25.10	23.89	–	27.27	25.02	23.38	22.24	21.19
Married^a^ (%)	40.23	73.70	80.37	79.55	76.51	73.30	38.73	56.56	63.66	61.48	57.34	50.70
Index person’s education^a^												
Basic (%)	59.15	50.16	45.63	47.40	53.42	64.03	48.84	56.04	52.16	53.58	60.66	71.57
Secondary (%)	28.62	34.93	36.16	32.89	30.23	26.12	32.32	32.11	33.63	31.75	28.74	23.13
Tertiary (%)	12.23	14.91	18.20	19.71	16.35	9.85	18.84	11.85	14.21	14.67	10.59	5.30
Partner’s education^a,b^												
Basic (%)	50.09	52.29	46.41	46.28	52.37	62.71	48.84	51.42	47.19	49.45	56.76	68.78
Secondary (%)	33.62	33.96	36.01	34.66	31.20	26.56	34.54	34.42	35.11	31.60	27.50	22.52
Tertiary (%)	16.29	13.75	17.59	19.05	16.43	10.73	16.62	14.16	17.70	18.96	15.74	8.61
Children’s education^a^												
Basic (%)		10.98	2.42	1.20	0.96	0.85		12.61	3.29	1.77	1.29	1.27
Secondary ≤2 yrs. (%)		30.70	21.76	18.16	18.39	20.33		30.63	23.22	20.40	21.24	24.33
Secondary > 2 yrs. (%)		16.13	15.63	14.76	16.12	17.79		15.45	14.96	14.39	15.86	17.76
Tertiary ≤2 yrs. (%)		17.24	21.48	20.58	20.10	20.60		17.15	21.48	21.11	20.57	21.18
Tertiary > 2 yrs. (%)		24.95	38.71	45.30	44.42	40.43		24.16	37.05	42.33	41.04	35.46

^a^At study entry; ^b^only married individuals included

### Number of children and health

**Fig. 1:**
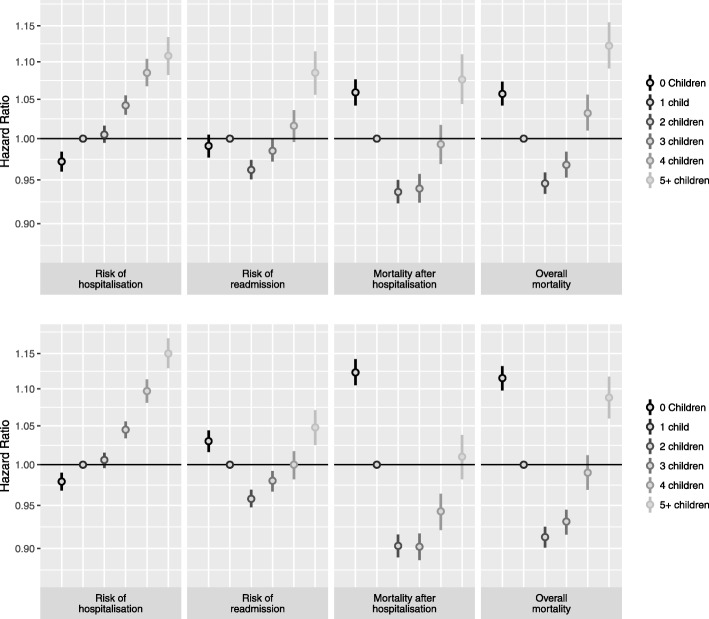
Hazard Ratios and 95% Confidence Intervals for the association of number of children with risk of hospitalisation, risk of re-admission, mortality after hospitalisation, and overall mortality. Upper panels men, lower panels women. Reference Category: Having one child. Models adjusted for index person’s education and income

The risk of hospitalisation was slightly lower among childless individuals compared to parents (Fig. 1). This difference disappeared after adjusting for partners’ education within the married subgroup (Additional file 1: Table S1). Among individuals with three or more children, the risk of hospitalisation was elevated compared to those with one or two children (Additional file 1: Table S1).

In contrast, a U-shaped relationship was found for number of children and risk of re-admission among women (Fig. 1, Additional file 1: Table S1). Men without children were not at higher risk of re-admission than men with one child, but fathers with five or more children had an elevated risk and those with two children a lower risk.

There was also a U-shaped association of number of children with mortality after hospitalisation among both men and women (Fig. 1, Additional file 1: Table S1) with a HR ranging from 0.90 to 1.12. The associations were observed among both men and women and remained after adjustment for confounders. Having two children was associated with the lowest mortality, and being childless or having 5 or more children the highest. The results for overall mortality were similar to the results for mortality after hospitalisation (Fig. 1, Additional file 1: Table S1).

### Children’s socioeconomic resources and parental health

**Fig. 2:**
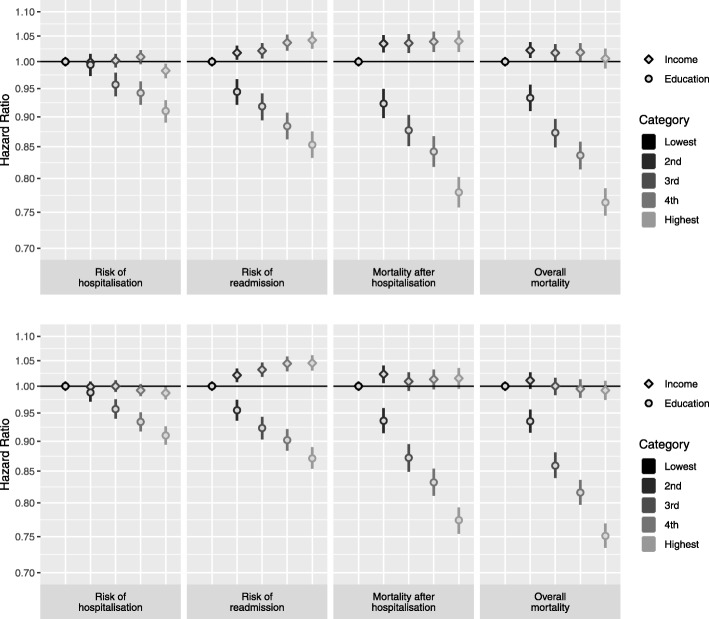
Hazard Ratios and 95% Confidence Intervals for the association of adult children’s education and income with risk of hospitalisation, risk of re-admission, mortality after hospitalisation, and overall mortality. Upper panels men, lower panels women. Reference Categories: Lowest income quintile and basic education. Models mutually adjusted for education and income and further adjusted for number of adult children, parents’ education and income and parental age at first birth

Higher offspring education was associated with a lower risk for all outcomes. This association persisted when accounting for number of children, and parent’s education, income, and age at first birth (Fig. 2). Children’s income was not associated with risk of hospitalisation or overall mortality (Fig. 2, Additional file 1: Table S2). However, a higher income of children was associated with a higher risk or re-admission and mortality after hospitalisation among parents, but effects were weaker than for education (Fig. 2, Additional file 1: Table S2).

The higher risk of hospitalisation among parents of three or more children remained after adjusting for adult children’s education and income (Additional file 1: Table S3). The lower mortality risk among parents with two or three children compared to those with one child was attenuated when adjusting for children’s education and income, particularly among mothers (Additional file 1: Table S3). This suggests that part of the association between parity and mortality is explained by the offspring’s educational level. However, those with five or more children remained at higher risk of adverse health outcomes even after adjustment.

### Predicted survival

Adjusted for potential confounders, the difference between proportions hospitalised by age 80 was 1.1 percentage points for men and 1.0 for women, in favour of childless individuals. The proportion of men with two children surviving up to age 80 was 2 percentage points higher than the proportion of childless men (Table 2). The corresponding number for women was 2.4 percentage points. By age 87, a difference of 3.4 percentage points for men and 4.4 percentage points for women was estimated.

**Table 2:** Predicted proportion of individuals not being hospitalised by age 80 years, not being re-admitted 2 years after hospitalisation, surviving 5 years after hospitalisation, and surviving to age 80 and 87 years, by number of children and children’s education level

Number of adult children	Men						Women					
	0	1	2	3	4	5+	0	1	2	3+	4	5+
Not hospitalised by age 80 years (%)	31.1	29.9	30.0	28.6	27.7	26.5	39.8	38.8	38.9	36.2	35.1	32.1
Not re-admitted 2 years after hospitalisation (%)	53.1	53.7	53.4	52.1	50.6	49.0	55.1	57.7	58.5	56.5	57.0	54.4
Surviving 5 years after hospitalisation (%)	73.6	75.2	75.4	76.0	74.2	70.6	80.2	81.3	83.9	83.6	83.3	81.7
Surviving to age 80 years (%)	73.9	74.7	75.9	75.7	74.7	71.5	83.7	84.7	86.1	85.6	85.2	83.1
Surviving to age 87 years (%)	41.2	42.5	44.6	44.2	42.5	37.4	55.9	58.2	61.3	60.1	59.4	54.7
	Men						Women					
Adult children’s education level	Basic	Sec. ≤2 yrs	Sec. > 2 yrs	Tert. ≤2 yrs	Tert. > 2 yrs		Basic	Sec. ≤2 yr	Sec. > 2 yrs	Tert. ≤2 yrs	Tert. > 2 yrs	
Not hospitalised by age 80 years (%)	27.9	29.5	31.2	31.6	33.0		38.0	38.7	39.7	41.0	41.6	
Not re-admitted 2 years after hospitalisation (%)	50.9	51.7	53.0	53.7	54.9		57.8	58.5	59.1	60.7	61.2	
Surviving 5 years after hospitalisation (%)	74.8	75.5	76.3	77.1	78.7		82.3	83.8	83.8	84.5	86.1	
Surviving to age 80 years (%)	73.1	75.1	76.3	77.3	79.2		84.4	85.7	86.5	87.4	88.3	
Surviving to age 87 years (%)	39.8	43.2	45.3	47.0	50.5		57.2	60.1	62.0	64.2	66.3	

Models were estimated for married individuals in the median birth cohort (and median year of hospitalisation) holding covariates (parental education and parental income in models for number of adult children, and, parental education and income, number of adult children, adult children’s income and parental age at first birth in models for adult children’s education level) at their mean. This can be interpreted as, for example, the predicted proportion surviving to age 80 years by number of children for individuals with basic education and income in the third quintile. Or as the predicted proportion surviving to age 80 years by adult children’s education level children for parents with basic education, income in the third quintile, with two adult children, children’s income in the third quintile and parental age at first birth of 25–30 years

The proportion hospitalised by age 80 was 5.1 percentage points higher for fathers of children with basic education than those of children with the highest education level. The according difference for mothers was 3.6 percentage points. The survival proportion up to age 80 for fathers of children with basic education was 6.1 percentage points lower than among fathers of children with the highest education level (Table 2). For women the respective difference was 3.9 percentage points. At age 87, the difference was 10.7 percentage points among men and 9.1 among women.

## Discussion

In this nationwide population-based cohort study we show that the association between parity and health in old age differs for different health outcomes, that is, between the risk of first hospitalisation and the risk of re-admission and mortality. While it is known that childless old people have higher mortality, this study indicates that this may result from a shorter survival once a disease has occurred. If hospitalisation is a proxy for disease onset, our results further suggest that childless individuals do not necessarily face an earlier disease onset. In addition, this study demonstrates that adult children’s educational level is important for both hospitalisation as well as survival, in fact more so than the number of children itself. Finally, our results indicate that offspring’s socioeconomic resources explain part of the curvilinear relationship between parity and later life mortality observed in this and in previous studies.

The finding that childless individuals have a higher mortality than parents is in line with previous studies [4]. Only among the small group of parents with five or more children (less than 3% of the study population), we found no reduced mortality compared to childless. A complex interplay of selection processes and biological and social effects of parity may result in an ‘optimal’ number of children for parental mortality [4]. To our knowledge, parity in relation to risk of hospitalisation, re-admission and mortality after hospitalisation has not previously been studied. As such, we cannot draw comparisons with previous work. It has been hypothesised that having children may promote healthier behaviours in parents [26], which may contribute to a delay in onset of disease. Our results do not support this hypothesis since the hospitalisation risk was not lower for parents than for childless individuals. It should be noted, however, that hospitalisations are not an ideal measure of disease onset and that the probability of hospitalisation may differ between parents and childless individuals regardless of disease status.

Having three or more children was associated with a greater risk of hospitalisation than having one child. This could be related to the balance between the positive effects of having children and selection processes defining the social background and composition of this group of parents [4] which results in a greater risk of hospitalisation but lower mortality. Alternatively, children could assist their parents with seeking inpatient care for existing health problems or with transport to the hospital.

Health selection into parenthood could be an explanation for differences in morbidity and mortality between parents and childless individuals, but is less likely that selection explains disparities in survival once a disease has occurred. The observed associations between having children and re-admission or survival after hospitalisation might thus suggest support from children to matter for prognosis after disease onset. However, these findings might also stem from a difference in the probability of becoming hospitalised. Adjusting for parents’ own education and income had almost no impact on the increased mortality of childless individuals. This, too, may indicate that selection into parenthood or an accumulation of disadvantage over the life-course is not the only force behind the survival disadvantage among individuals without children, but that support from adult children also matters.

Consistent with previous studies we found adult children’s education to be more closely related to parental mortality than children’s income [14, 22]. Building on this earlier work, our results show that education of adult children is associated with both disease onset in parents and with survival after ill health has begun. Even though the children’s socioeconomic resources seem to matter more for health in old age, the associations with number of adult children remained after adjusting for children’s education and income. This suggests that the number of children, independent of their socioeconomic resources, matters for the survival of their parents, but perhaps through different mechanisms.

In Sweden, elderly care is universally available and almost fully subsidised. Differences in access to health and elderly care are therefore less likely to explain the results than in other contexts. However, a publically financed health care system also comes with limitations of resources and a slim lined organisation that could increase differences between individuals with and without family support. Although cohabitation of adult children with their parents is uncommon in Sweden, the majority of parents has contact with their children at least once per week [27, 28]. Moreover, adult children often become care-givers for their aging parents [11]. Still, it is possible that children and their socioeconomic resources play an even larger role for their parents’ health in countries in which care is not publically financed. In order to gain further insight in the relationship between childlessness, health, and survival in old age we suggest future studies continue looking into mechanisms linking adult children to parental health and focus, for instance, on the different types of support that could affect health in old age. In an era in which childlessness is increasing [29] this is important to inform public health policies and reduce survival inequalities.

### Strengths and limitations

This large population-based cohort study has complete information since it is based on national register data, and therefore a low risk of exposure and outcome misclassification or selection bias. It is the first study, to our knowledge, to examine and compare the risk of hospitalisation, re-admission, and mortality after hospitalisation among parents compared to childless individuals. Nonetheless, there are some limitations of the work. It could be argued that hospitalisations are a poor proxy for disease onset. For example, some people could experience disease onset without being admitted to hospital, while other people may be admitted to hospital for relatively minor difficulties. However, requiring hospitalisation for at least two nights ensured some degree of severity, and the publically financed healthcare in Sweden ensures equal chances of being admitted for everyone. Thus, we believe both specificity (all with a severe condition are hospitalised) and sensitivity (the hospitalised are truly ill) are high given the definition of onset of severe disease. It could further be argued that adult children’s socioeconomic resources are proxy measures of their parent’s socioeconomic position, rather than an independent exposure. However, if this was the case we would expect similar associations with both adult children’s education and income. The fact that we observed associations with parental outcomes for adult children’s education but not income suggests that the mechanisms driving these associations are related to adult children’s education per se. It should, however, be noted that the inequality in income is rather low in Sweden compared to other countries. Adult children’s income might perhaps play a larger role for parental health in other contexts.

## Conclusions

This nationwide study confirms that the presence and number of adult children are associated with mortality in old age, with childless individuals being at greatest risk. It adds to previous knowledge showing that the benefit of having children for health in old age seems to arise once individuals have become ill rather than before. In times of rising childlessness and increasing longevity, our findings raise concern about potential care deficits of older childless individuals, particularly those affected by disease. Our results further indicate the potential importance of resources provided by highly educated children for parental health and mortality in old age.

## Additional files

Additional file 1: **Table S1.** Association of number of adult children with overall mortality, risk of hospitalisation, risk of readmission, and mortality after hospitalisation. Data source same as in the main analyses, all individuals born 1920–1940 alive and residing in Sweden at age 70 and their children, collected from national registers. **Table S2.** Association of adult children’s education and income with overall mortality, risk of hospitalisation, risk of readmission, and mortality after hospitalisation among parents. Data source same as in the main analyses. Data source same as in the main analyses, all individuals born 1920–1940 alive and residing in Sweden at age 70 and their children, collected from national registers. **Table S3.** Association of number of adult children with overall mortality, risk of hospitalisation, risk of readmission, and mortality after hospitalisation among parents accounting for adult children’s education and income. Data source same as in the main analyses. Data source same as in the main analyses, all individuals born 1920–1940 alive and residing in Sweden at age 70 and their children, collected from national registers. (DOCX 49 kb)

## Abbreviations

CI: Confidence interval
HR: Hazard ratio

## Footnotes

1 For index persons for whom weights were not available, we applied weights for individuals living alone or couples depending on their marital status (since elderly individuals are likely to either live alone or with their partner, but not with children). If weights for children were missing, we did not apply any weights.
