# Supplementary material for: The role of children and their socioeconomic resources for the risk of hospitalisation and mortality – a nationwide register-based study of the total Swedish population over the age 70
Source: BMC Geriatr. 2019 Apr 23;19:114. doi: 10.1186/s12877-019-1134-y (PMC6480801; doi:10.1186/s12877-019-1134-y)
Supplement: Supplementary file 1 — Table S1. Association of number of adult children with overall mortality, risk of hospitalisation, risk of readmission, and mortality after hospitalisation. Data source same as in the main analyses, all individuals born 1920–1940 alive and residing in Sweden at age 70 and their children, collected from national registers. Table S2. Association of adult children’s education and income with overall mortality, risk of hospitalisation, risk of readmission, and mortality after hospitalisation among parents. Data source same as in the main analyses. Data source same as in the main analyses, all individuals born 1920–1940 alive and residing in Sweden at age 70 and their children, collected from national registers. Table S3. Association of number of adult children with overall mortality, risk of hospitalisation, risk of readmission, and mortality after hospitalisation among parents accounting for adult children’s education and income. Data source same as in the main analyses. Data source same as in the main analyses, all individuals born 1920–1940 alive and residing in Sweden at age 70 and their children, collected from national registers. (DOCX 49 kb) [file 12877_2019_1134_MOESM1_ESM.docx]

## Supplementary table 1. Association of number of adult children with risk of hospitalisation, risk of readmission, mortality after hospitalisation, and overall mortality

|  |  | **Men** | | | **Women** | | |
| --- | --- | --- | --- | --- | --- | --- | --- |
|  | **No.**  **Children** | **M1**  HR [95%-CI] | **M2**  HR [95%-CI] | **M3**  HR [95%-CI] | **M1**  HR [95%-CI] | **M2**  HR [95%-CI] | **M3**  HR [95%-CI] |
| Risk of  hospitalisation |  | n=401,997 | n=401,997 | n=283,702 | n=488,547 | n=488,547 | n=278,958 |
|  | 0 | 0.98 [0.97,0.99] | 0.97 [0.96,0.98] | 1.00 [0.98,1.01] | 0.97 [0.96,0.98] | 0.98 [0.97,0.99] | 1.00 [0.98,1.01] |
|  | 1 | 1.00 [1.00,1.00] | 1.00 [1.00,1.00] | 1.00 [1.00,1.00] | 1.00 [1.00,1.00] | 1.00 [1.00,1.00] | 1.00 [1.00,1.00] |
|  | 2 | 1.00 [0.99,1.01] | 1.01 [1.00,1.02] | 1.01 [1.00,1.02] | 1.00 [0.99,1.01] | 1.01 [1.00,1.02] | 1.00 [0.99,1.02] |
|  | 3 | 1.04 [1.02,1.05] | 1.04 [1.03,1.05] | 1.05 [1.03,1.06] | 1.05 [1.03,1.06] | 1.04 [1.03,1.06] | 1.04 [1.03,1.06] |
|  | 4 | 1.09 [1.07,1.11] | 1.09 [1.07,1.10] | 1.09 [1.07,1.11] | 1.11 [1.09,1.12] | 1.10 [1.08,1.11] | 1.10 [1.07,1.12] |
|  | 5+ | 1.13 [1.10,1.16] | 1.11 [1.08,1.13] | 1.12 [1.09,1.15] | 1.18 [1.15,1.20] | 1.15 [1.13,1.17] | 1.16 [1.13,1.19] |
|  |  |  |  |  |  |  |  |
| Risk of  readmission |  | n=279,328 | n=279,328 | n=199,240 | n=332,369 | n=332,369 | n=187,506 |
|  | 0 | 1.01 [0.99,1.02] | 0.99 [0.98,1.01] | 1.00 [0.98,1.02] | 1.02 [1.00,1.03] | 1.03 [1.02,1.04] | 1.03 [1.01,1.05] |
|  | 1 | 1.00 [1.00,1.00] | 1.00 [1.00,1.00] | 1.00 [1.00,1.00] | 1.00 [1.00,1.00] | 1.00 [1.00,1.00] | 1.00 [1.00,1.00] |
|  | 2 | 0.96 [0.94,0.97] | 0.96 [0.95,0.97] | 0.96 [0.94,0.97] | 0.95 [0.94,0.97] | 0.96 [0.95,0.97] | 0.95 [0.94,0.96] |
|  | 3 | 0.98 [0.97,0.99] | 0.99 [0.97,1.00] | 0.98 [0.96,0.99] | 0.98 [0.97,0.99] | 0.98 [0.97,0.99] | 0.98 [0.96,0.99] |
|  | 4 | 1.02 [1.00,1.04] | 1.02 [1.00,1.04] | 1.01 [0.98,1.03] | 1.01 [0.99,1.03] | 1.00 [0.98,1.02] | 1.00 [0.98,1.02] |
|  |  |  |  |  |  |  |  |
| Mortality after  hospitalisation |  | n=279,328 | n=279,328 | n=199,240 | n=332,369 | n=332,369 | n=187,506 |
|  | 0 | 1.09 [1.07,1.10] | 1.06 [1.04,1.08] | 1.07 [1.04,1.09] | 1.10 [1.08,1.11] | 1.12 [1.10,1.14] | 1.12 [1.09,1.14] |
|  | 1 | 1.00 [1.00,1.00] | 1.00 [1.00,1.00] | 1.00 [1.00,1.00] | 1.00 [1.00,1.00] | 1.00 [1.00,1.00] | 1.00 [1.00,1.00] |
|  | 2 | 0.93 [0.91,0.94] | 0.94 [0.92,0.95] | 0.94 [0.92,0.96] | 0.90 [0.89,0.91] | 0.90 [0.89,0.92] | 0.91 [0.89,0.92] |
|  | 3 | 0.93 [0.92,0.95] | 0.94 [0.92,0.96] | 0.94 [0.92,0.96] | 0.90 [0.89,0.92] | 0.90 [0.89,0.92] | 0.90 [0.88,0.92] |
|  | 4 | 1.00 [0.98,1.03] | 0.99 [0.97,1.02] | 0.98 [0.95,1.01] | 0.96 [0.94,0.98] | 0.94 [0.92,0.96] | 0.94 [0.91,0.97] |
|  | 5+ | 1.12 [1.09,1.16] | 1.08 [1.04,1.11] | 1.08 [1.04,1.12] | 1.06 [1.03,1.09] | 1.01 [0.98,1.04] | 1.01 [0.97,1.05] |
|  |  |  |  |  |  |  |  |
| Overall  mortality |  | n=401,997 | n=401,997 | n=283,702 | n=488,547 | n=488,547 | n=278,958 |
|  | 0 | 1.09 [1.07,1.11] | 1.06 [1.04,1.07] | 1.07 [1.05,1.09] | 1.08 [1.07,1.10] | 1.11 [1.10,1.13] | 1.11 [1.09,1.14] |
|  | 1 | 1.00 [1.00,1.00] | 1.00 [1.00,1.00] | 1.00 [1.00,1.00] | 1.00 [1.00,1.00] | 1.00 [1.00,1.00] | 1.00 [1.00,1.00] |
|  | 2 | 0.93 [0.92,0.95] | 0.95 [0.93,0.96] | 0.95 [0.94,0.97] | 0.91 [0.90,0.92] | 0.91 [0.90,0.92] | 0.92 [0.90,0.94] |
|  | 3 | 0.96 [0.94,0.97] | 0.97 [0.95,0.98] | 0.97 [0.96,0.99] | 0.93 [0.92,0.95] | 0.93 [0.92,0.95] | 0.94 [0.92,0.96] |
|  | 4 | 1.04 [1.02,1.07] | 1.03 [1.01,1.06] | 1.03 [1.00,1.06] | 1.01 [0.99,1.04] | 0.99 [0.97,1.01] | 0.99 [0.96,1.02] |
|  | 5+ | 1.18 [1.15,1.21] | 1.12 [1.09,1.16] | 1.13 [1.10,1.17] | 1.15 [1.12,1.18] | 1.09 [1.06,1.12] | 1.10 [1.06,1.14] |

M1: Crude, M2: adjusted for education and income of the index person, M3: additionally adjusted for the index persons partner’s education (M3 only includes index persons with a partner)

## Supplementary table 2. Association of adult children’s education and income with risk of hospitalisation, risk of readmission, mortality after hospitalisation, and overall mortality among parents

|  | **Men** | | | | **Women** | | | |
| --- | --- | --- | --- | --- | --- | --- | --- | --- |
|  | **Risk of**  **hospitalisation**  HR [95% CI] | **Risk of readmission**  HR [95% CI] | **Mortality after**  **hospitalisation**  HR [95% CI] | **Overall mortality**  HR [95% CI] | **Risk of**  **hospitalisation**  HR [95% CI] | **Risk of readmission**  HR [95% CI] | **Mortality after**  **hospitalisation**  HR [95% CI] | **Overall mortality**  HR [95% CI] |
|  | n=251,538 | n=176,433 | n=176,433 | n=251,538 | n=249,699 | n=167,543 | n=167,543 | n=249,699 |
| Child education |  |  |  |  |  |  |  |  |
| Basic | 1.00 [1.00,1.00] | 1.00 [1.00,1.00] | 1.00 [1.00,1.00] | 1.00 [1.00,1.00] | 1.00 [1.00,1.00] | 1.00 [1.00,1.00] | 1.00 [1.00,1.00] | 1.00 [1.00,1.00] |
| Sec. ≤2 yrs | 0.99 [0.97,1.02] | 0.95 [0.92,0.98] | 0.93 [0.90,0.96] | 0.94 [0.91,0.97] | 0.98 [0.95,1.00] | 0.96 [0.93,0.99] | 0.94 [0.91,0.98] | 0.94 [0.91,0.97] |
| Sec. >2 yrs | 0.96 [0.93,0.98] | 0.92 [0.90,0.95] | 0.88 [0.85,0.91] | 0.88 [0.85,0.90] | 0.95 [0.93,0.98] | 0.94 [0.91,0.96] | 0.89 [0.86,0.93] | 0.88 [0.85,0.91] |
| Tertiary ≤2 yrs | 0.94 [0.91,0.96] | 0.88 [0.86,0.91] | 0.84 [0.82,0.87] | 0.84 [0.81,0.86] | 0.92 [0.89,0.94] | 0.91 [0.89,0.94] | 0.85 [0.82,0.88] | 0.83 [0.80,0.86] |
| Tertiary >2 yrs | 0.91 [0.88,0.93] | 0.86 [0.84,0.89] | 0.78 [0.76,0.81] | 0.76 [0.74,0.79] | 0.90 [0.88,0.92] | 0.88 [0.86,0.91] | 0.79 [0.76,0.82] | 0.77 [0.74,0.79] |
| Child income |  |  |  |  |  |  |  |  |
| 1st quintile | 1.00 [1.00,1.00] | 1.00 [1.00,1.00] | 1.00 [1.00,1.00] | 1.00 [1.00,1.00] | 1.00 [1.00,1.00] | 1.00 [1.00,1.00] | 1.00 [1.00,1.00] | 1.00 [1.00,1.00] |
| 2nd quintile | 1.00 [0.98,1.01] | 1.01 [1.00,1.03] | 1.02 [1.00,1.04] | 1.01 [1.00,1.03] | 1.00 [0.98,1.01] | 1.02 [1.00,1.03] | 1.03 [1.00,1.05] | 1.02 [1.00,1.04] |
| 3rd quintile | 1.00 [0.99,1.02] | 1.02 [1.00,1.04] | 1.02 [1.00,1.04] | 1.00 [0.98,1.02] | 1.00 [0.99,1.02] | 1.03 [1.01,1.05] | 1.01 [0.99,1.04] | 1.01 [0.98,1.03] |
| 4th quintile | 1.01 [0.99,1.02] | 1.03 [1.01,1.05] | 1.04 [1.01,1.06] | 1.01 [0.99,1.03] | 0.99 [0.98,1.01] | 1.05 [1.03,1.07] | 1.02 [1.00,1.05] | 1.01 [0.98,1.03] |
| 5th quintile | 0.98 [0.97,1.00] | 1.04 [1.02,1.06] | 1.03 [1.01,1.06] | 1.00 [0.98,1.02] | 0.98 [0.97,1.00] | 1.04 [1.02,1.06] | 1.03 [1.00,1.05] | 1.00 [0.98,1.03] |

All models mutually adjusted for adult children’s education and income level, and further adjusted for number of adult children, the index person’s education and income, parental age at first birth and partner’s education (N.B. models only include index persons with a partner)

## Supplementary table 3. Association of number of adult children with overall mortality, risk of hospitalisation, risk of readmission, and mortality after hospitalisation among parents accounting for adult children’s education and income

|  |  | **Men** | | | | **Women** | | |
| --- | --- | --- | --- | --- | --- | --- | --- | --- |
|  | **No. Children** | **M1**  HR [95%-CI] | **M2**  HR [95%-CI] | | **M3**  HR [95%-CI] | **M1**  HR [95%-CI] | **M2**  HR [95%-CI] | **M3**  HR [95%-CI] |
|  |  | n=322,143 | | n=322,143 | n=251,538 | n=413,086 | n=413,086 | n=249,699 |
| Overall  mortality | 1 | 1.00 [1.00,1.00] | 1.00 [1.00,1.00] | | 1.00 [1.00,1.00] | 1.00 [1.00,1.00] | 1.00 [1.00,1.00] | 1.00 [1.00,1.00] |
|  | 2 | 0.95 [0.93,0.96] | 0.96 [0.95,0.98] | | 0.97 [0.96,0.99] | 0.91 [0.90,0.93] | 0.94 [0.93,0.95] | 0.94 [0.92,0.96] |
|  | 3 | 0.97 [0.95,0.99] | 0.98 [0.97,1.00] | | 0.99 [0.97,1.01] | 0.93 [0.92,0.95] | 0.96 [0.94,0.97] | 0.96 [0.94,0.98] |
|  | 4 | 1.03 [1.01,1.06] | 1.04 [1.02,1.07] | | 1.04 [1.01,1.07] | 0.99 [0.97,1.01] | 1.01 [0.99,1.03] | 1.01 [0.98,1.04] |
|  | 5+ | 1.13 [1.09,1.16] | 1.12 [1.09,1.16] | | 1.14 [1.10,1.18] | 1.09 [1.06,1.12] | 1.10 [1.07,1.13] | 1.10 [1.06,1.15] |
|  |  |  |  | |  |  |  |  |
| Risk of  hospitalisation | 1 | 1.00 [1.00,1.00] | 1.00 [1.00,1.00] | | 1.00 [1.00,1.00] | 1.00 [1.00,1.00] | 1.00 [1.00,1.00] | 1.00 [1.00,1.00] |
|  | 2 | 1.01 [1.00,1.02] | 1.01 [1.00,1.02] | | 1.02 [1.01,1.03] | 1.01 [1.00,1.02] | 1.01 [1.00,1.02] | 1.01 [1.00,1.03] |
|  | 3 | 1.04 [1.03,1.06] | 1.05 [1.03,1.06] | | 1.05 [1.04,1.07] | 1.05 [1.03,1.06] | 1.05 [1.04,1.06] | 1.05 [1.03,1.06] |
|  | 4 | 1.09 [1.07,1.10] | 1.09 [1.07,1.11] | | 1.09 [1.07,1.11] | 1.10 [1.08,1.11] | 1.09 [1.08,1.11] | 1.09 [1.07,1.12] |
|  | 5+ | 1.11 [1.08,1.13] | 1.10 [1.07,1.13] | | 1.11 [1.08,1.14] | 1.15 [1.13,1.17] | 1.14 [1.11,1.16] | 1.15 [1.12,1.18] |
|  |  | n=224200 | | | n=176433 | n=280937 | | n=167543 |
| Risk of  readmission | 1 | 1.00 [1.00,1.00] | 1.00 [1.00,1.00] | | 1.00 [1.00,1.00] | 1.00 [1.00,1.00] | 1.00 [1.00,1.00] | 1.00 [1.00,1.00] |
|  | 2 | 0.96 [0.95,0.97] | 0.97 [0.96,0.99] | | 0.97 [0.95,0.98] | 0.96 [0.95,0.97] | 0.97 [0.96,0.98] | 0.96 [0.94,0.97] |
|  | 3 | 0.99 [0.97,1.00] | 1.00 [0.98,1.01] | | 0.99 [0.97,1.01] | 0.98 [0.97,0.99] | 0.99 [0.98,1.00] | 0.99 [0.97,1.00] |
|  | 4 | 1.02 [1.00,1.04] | 1.03 [1.01,1.05] | | 1.02 [1.00,1.05] | 1.00 [0.98,1.02] | 1.01 [0.99,1.03] | 1.00 [0.98,1.03] |
|  | 5+ | 1.08 [1.06,1.11] | 1.10 [1.06,1.13] | | 1.10 [1.07,1.14] | 1.05 [1.03,1.07] | 1.05 [1.03,1.07] | 1.04 [1.01,1.08] |
|  |  |  |  | |  |  |  |  |
| Mortality after  hospitalisation | 1 | 1.00 [1.00,1.00] | 1.00 [1.00,1.00] | | 1.00 [1.00,1.00] | 1.00 [1.00,1.00] | 1.00 [1.00,1.00] | 1.00 [1.00,1.00] |
|  | 2 | 0.94 [0.92,0.95] | 0.95 [0.94,0.97] | | 0.95 [0.94,0.97] | 0.90 [0.89,0.92] | 0.93 [0.91,0.94] | 0.93 [0.91,0.94] |
|  | 3 | 0.94 [0.93,0.96] | 0.95 [0.93,0.97] | | 0.95 [0.93,0.97] | 0.90 [0.89,0.92] | 0.93 [0.91,0.94] | 0.93 [0.90,0.95] |
|  | 4 | 0.99 [0.97,1.02] | 1.00 [0.97,1.03] | | 0.99 [0.96,1.02] | 0.94 [0.92,0.97] | 0.96 [0.94,0.99] | 0.96 [0.92,0.99] |
|  | 5+ | 1.08 [1.04,1.11] | 1.07 [1.04,1.11] | | 1.08 [1.04,1.12] | 1.01 [0.98,1.04] | 1.03 [1.00,1.06] | 1.02 [0.98,1.06] |

M1: Adjusted for parents’ income and education only (corresponding to M2 in supplementary table 1 to facilitate comparison of results), M2: additionally adjusted for adult children’s education and income and parental age at first birth, M3: additionally adjusted for the index persons partner’s education (M3 only includes index persons with a partner)
